# The computationally predicted drug-likeness, pharmacokinetics properties, medicinal chemistry parameters, and toxicity properties of *Cucurbita maxima *compounds.

**DOI:** 10.12688/f1000research.127126.1

**Published:** 2022-10-31

**Authors:** Ryman Shoko

**Affiliations:** 1 Biology, Chinhoyi University of Technology, Chinhoyi, Zimbabwe

**Keywords:** Cucurbita maxima, medicinal plants, druglikeness, natural products, pharmacoinformatics

## Abstract

Natural compounds are increasingly becoming an important source of drug leads for computer-aided drug design approaches. *Cucurbita maxima* has been observed to have medicinal properties and can, therefore, be a potential source of novel drug leads. However, before compounds can be synthesized in the lab for tests, modern approaches require that the candidate compounds be screened for drug-likeness characteristics and toxicity, among others. In this work, the computational tools, SwissADME and DataWarrior were used to screen *C. maxima* compounds for their potential consideration as drug leads. A total of 130 compounds, downloaded from the LOTUS natural products database, were computationally analysed. The data set presented in this work will be useful to researchers searching for novel drug leads based on natural compounds.

## Introduction

Natural products (NP), such as plants and their extracts, have been used to cure diseases in humans and livestock since ancient times ( Daina *et al*., 2017; Greenwell & Rahman, 2015). In modern computer-aided drug design approaches, NPs are considered to be a significant foundation for drug discovery due to their diverse chemical components and their often-unique biomedical properties ( Süntar, 2020). Among their unique properties, the NPs are often rich in stereogenic centres and occupy portions of the chemical space that is usually not covered by most synthetic drugs ( Marxer *et al*., 2012).

*Cucurbita maxima* (commonly known as giant pumpkin) is rich in phenolics, tannins, flavonoids, alkaloids, saponins, terpenoids, carbohydrates and proteins ( Salehi *et al*., 2019; Sorescu *et al*., 2020). For centuries, extracts from different parts of the plant have been used to treat various diseases such as intestinal infections, renal failure, hyperplasia, constipation, and parasite infestation ( Menendez-Baceta *et al*., 2014; Kujawska & Pieroni, 2015; Mahomoodally *et al*., 2016; Mtemeli *et al*. 2021). Thus, CADD approaches can be applied to investigate the potential of some compounds from this plant to act as drug leads. Before synthesising a compound in the laboratory for testing, modern computational approaches require that the compounds be computationally screened for drug-likeness and potential toxicity.

The standard method to evaluate drug-likeness of a compound is to assess compliance to Lipinski's Rule of Five ( Lipinski *et al*., 1997), which covers the molecular weight, numbers of hydrophilic groups and hydrophobicity. This data note presents a list of *C. maxima* natural compounds and their computationally calculated data on drug-likeness characteristics, pharmacokinetics, medicinal chemistry parameters and predicted toxicity. Toxicity predictions are important because substructures with known toxic, teratogenic or mutagenic properties negatively affects the usefulness of a designed drug. With data produced in this work, researchers can better predict which *C. maxima* compounds have a better chance of succeeding throughout all stages of clinical trials, through to drug approval.

## Materials and methods

To create a library of *C. maxima* natural compounds, the term 'Cucurbita maxima' was entered into the search box of the Lotus Natural Compounds Database ( https://lotus.naturalproducts.net/). The search returned 130 natural products. A file containing the 130 compounds in the structure-data file (SDF) format was downloaded and then fed into BIOVIA Discovery Studio *v21.*1.0.20298, RRID:SCR_015651 to get the molecular structures in the corresponding simplified molecular-input line-entry system (SMILE) format. The SMILEs were then used to calculate the various properties of the compounds using the SwissADME ( Daina *et al*., 2017) web tool and the DataWarrior v5.5.0 ( Sander *et al*., 2015) software.

## Dataset validation and limitations

An inherent limitation of computational prediction of drug-likeness is the lack of validated datasets of drugs and non-drugs. Therefore, the classification presented here is solely based on the similarity of structure of the compounds to known drugs. Also compounds from completely new classes are likely to be wrongly classified. Another important limitation of computationally predicted drug-likeness is that it does not predict the biological/pharmacological activity of a compound. Wet bench methods are required to validate the biological/pharmacological activity.

In summary, the dataset presented here will probably be most useful in lead discovery where they could be used for prioritizing compounds for synthesis or for purchasing from external suppliers.

## Data Availability

Harvard Dataverse: Underlying data for ‘The Computationally predicted drug-likeness, pharmacokinetics properties, medicinal chemistry parameters, and toxicity properties of Cucurbita maxima compounds’. https://doi.org/10.7910/DVN/4ISBWI ( Shoko, 2022). This project contains the following underlying data:
• Data file 1. (Druglikeness Properties of C. maxima natural compounds.) • Data file 2. (Medicinal Chemistry Properties of C. maxima natural compounds.) • Data file 3. (Pharmacokinetics Properties of C. maxima compounds.) • Data file 4. (Toxicity Properties of C. maxima compounds.) Data are available under the terms of the CC0 1.0 Universal (CC0 1.0) Public Domain Dedication.
