# Supplementary material for: The computationally predicted drug-likeness, pharmacokinetics properties, medicinal chemistry parameters, and toxicity properties of Cucurbita maxima compounds
Source: F1000Res. 2022 Oct 31;11:Chem Inf Sci-1234. [Version 1] doi: 10.12688/f1000research.127126.1 (PMC11058453; doi:10.12688/f1000research.127126.1)
Supplement: Peer review history

# Reviewer report: Reviewer response for version 1

**DOI:** 10.5256/f1000research.139601.r262625

**Published:** 2024-04-29

**Authors:** Gurudutt Dubey

**Affiliations:** 1 Indian Institute of Technology (IIT), Palaj, Gujarat, India; 2 Universidad Catolica de Valencia San Vicente Martir (Ringgold ID: 83140), Valencia, Valencian Community, Spain

This data not summaries the computationally predicted ADME and toxicity parameters of *Cucurbita maxima.* Author has used SwissADME and DataWarrior tools to generate the database. Introduction is well written but incorporation of some latest references is recommended. At some places, full form of the abbreviations are missing for e.g. CADD. I will suggest to incorporate ADME and toxicity parameters calculated using other tools than SwissADME as well. It will be helpful to understand the variability in the parameters calculated using various tools.

Are sufficient details of methods and materials provided to allow replication by others?

Yes

Is the rationale for creating the dataset(s) clearly described?

Yes

Are the datasets clearly presented in a useable and accessible format?

Yes

Are the protocols appropriate and is the work technically sound?

Yes

Reviewer Expertise:

Drug Discovery, Medicinal Chemistry, Computer-aided drug design, computational chemistry, RNA biology

I confirm that I have read this submission and believe that I have an appropriate level of expertise to confirm that it is of an acceptable scientific standard.

# Reviewer report: Reviewer response for version 1

**DOI:** 10.5256/f1000research.139601.r230597

**Published:** 2024-02-21

**Authors:** Edgar López-López

**Affiliations:** 1 Department of Chemistry and Graduate Program in Pharmacology, Center for Research and Advanced Studies of the National Polytechnic Institute,, Mexico City, Mexico

Dear author,

 I consider this "data note" to be interesting and transcendent. This work presents a dataset of properties of pharmaceutical interest of molecules present in Curcubita maxima, which could be of interest to those research groups that plan to isolate, synthesize, or acquire any of these molecules for subsequent trials. However, I consider that some minor comments should be resolved :

 1. Introduction section: About the sentence "... occupy portions of the chemical space that is usually not covered by most synthetic drugs". I suggest adding most recent references that support it. I share with you examples that could be useful: (Saldívar-G et al, 2019)[Ref 1] ,(Sorokina M  et al, 2021) [Ref 2], (Medina-Franco JL et al, 2022) [Ref 3], (López-Pérez K

 et al, 2023) [Ref 4]

 2. Introduction section: The abbreviation "CADD" could be unclear for "non-expert readers", I suggest adding the explicit significance of this.

 3. Please change "SMILE" to "SMILES".

 4. I suggest citing this "technology evaluation" article that complements the original Data Warrior reference: (López-López et al, 2019) [Ref 5]

 5. Please, add a reference that supports the sentence "Another important limitation of computationally predicted drug-likeness is that it does not predict the biological/pharmacological activity of a compound. "

 6. Scientific names (e.g. Curcubita maxima) must be written using italic style.

Are sufficient details of methods and materials provided to allow replication by others?

Yes

Is the rationale for creating the dataset(s) clearly described?

Yes

Are the datasets clearly presented in a useable and accessible format?

Yes

Are the protocols appropriate and is the work technically sound?

Partly

Reviewer Expertise:

Cheminformatics, Natural products, Drug design, Drug development, Computer-aided drug design.

I confirm that I have read this submission and believe that I have an appropriate level of expertise to confirm that it is of an acceptable scientific standard.

## References

[1] : Chemical Space and Diversity of the NuBBE Database: A Chemoinformatic Characterization. *J Chem Inf Model* .2019;59(1) : 10.1021/acs.jcim.8b00619 74-85 10.1021/acs.jcim.8b00619 30508485

[2] : COCONUT online: Collection of Open Natural Products database. *J Cheminform* .2021;13(1) : 10.1186/s13321-020-00478-9 2 10.1186/s13321-020-00478-9 33423696 PMC7798278

[3] : Chemical Multiverse: An Expanded View of Chemical Space. *Mol Inform* .2022;41(11) : 10.1002/minf.202200116 e2200116 10.1002/minf.202200116 35916110 PMC9787733

[4] : Sampling and Mapping Chemical Space with Extended Similarity Indices. *Molecules* .2023;28(17) : 10.3390/molecules28176333 10.3390/molecules28176333 37687162 PMC10489020

[5] : DataWarrior: an evaluation of the open-source drug discovery tool. *Expert Opin Drug Discov* .2019;14(4) : 10.1080/17460441.2019.1581170 335-341 10.1080/17460441.2019.1581170 30806519
